# Patients' knowledge about the uses, risks, and beliefs surrounding the regulation and safety of *Cannabis sativa L.* in Peru

**DOI:** 10.1016/j.heliyon.2024.e27068

**Published:** 2024-03-21

**Authors:** José F. Ramírez-Méndez, Pedro Wong-Salgado, Peter Gámez, Pedro Solis, Jeel Moya-Salazar

**Affiliations:** a CANNAVITAL Clínica Especializada en Terapias con Cannabinoides, Lima, Peru; b Centro de Estudios del Cannabis, Lima, Peru; c Department of Medicine, Essalud Hospital Jorge Voto Bernales Corpancho, Lima, Peru; d Graduate School, Universidad Alas Peruanas, Lima, Peru; e Digital Transformation Center, Universidad Norbert Wiener, Lima, Peru

**Keywords:** Medical marijuana, Knowledge, Cannabis, Survey, Peru, Marijuana, Assessment

## Abstract

In recent decades, there has been a significant rise in the utilization of medical cannabis. Understanding the various facets surrounding its usage and regulation is paramount for patients, health professionals, and policymakers. This cross-sectional study conducted at the CANNAVITAL clinic in Lima, Peru aimed to assess the basic knowledge, attitudes towards use, and beliefs regarding regulation and safety of *Cannabis sativa L.* Among 86 patients (mean age: 41 ± 7.8 years; 53.4% women) actively receiving medical cannabis treatment for at least one year, we utilized the 22-item KUC-22 questionnaire to evaluate their understanding of cannabis, risk factors, legislation, medical and recreational use. The average duration of medical cannabis use was 3 ± 1.2 years. Results showed that 60.2% of patients were familiar with different forms of cannabis consumption, while 77.3% recognized the importance of product quality. Chronic pain, nausea and vomiting (each 23.9%) were the primary conditions treated with cannabis, followed by cancer and epilepsy (both 21.6%). A significant correlation was found between knowledge and cannabis use (p < 0.001). Furthermore, 92% of patients believed that a medical prescription was necessary, while 84.1% engaged in self-medication practices. Concerning perceptions, 69.3% of participants perceived psychological dependence from long-term cannabis use, and 65.9% believed it could cause health damage. Significant correlations were observed between the perception of risk factors, knowledge of legislation, and cannabis use (p < 0.001). In conclusion, Peruvian patients exhibited a high level of knowledge regarding the risks, uses, and regulatory framework surrounding *Cannabis sativa L.* These findings provide valuable insights into patients' perspectives on medical cannabis, offering important information for medical providers and researchers aiming to enhance cannabinoid-based therapies.

## 1 Introduction

The global pharmaceutical sector is increasingly acknowledging the therapeutic potential of cannabis and the health advantages provided by its active compounds, known as cannabinoids. These advantages encompass the relief of chronic pain, muscle spasticity in multiple sclerosis, nausea and vomiting induced by chemotherapy, refractory epilepsy, depression, anxiety, sleep disorders, and rheumatic diseases [1,2]. Consequently, numerous countries worldwide are enacting regulations to govern the medicinal use of cannabis. With already more than 30 countries implementing regulations to facilitate cultivation, processing, research, and commercialization [3,4], the cannabis industry is anticipated to burgeon into a global market estimated at $194 billion within the next few years. This burgeoning market is expected to cater to an estimated consumer base exceeding 300 million individuals across diverse presentations and sectors [5].

In Peru, the regulation of cannabis and its derivatives for therapeutic and medicinal purposes falls under the purview of Law 30,681, officially enacted on November 17, 2017 [6]. To oversee this regulatory framework, the Ministry of Health (MINSA), specifically the General Directorate of Medicines, Supplies, and Drugs (DIGEMID), has introduced a mandatory virtual patient registration module. This module is a prerequisite for the sale and dispensation of cannabis products within pharmaceutical establishments. Additionally, the law delineates three distinct types of licenses: a license tailored for scientific research conducted by universities and research institutes specializing in agriculture and health, a production license designated for public entities and certified laboratories, and an importation and/or commercialization license [7].

The regulatory framework for medical cannabis has continued in Peru and on February 23, 2019, the regulation of Law 30,681 was approved, where the difference between psychoactive cannabis (any cannabis plant that possesses more than 1% of Δ9-tetrahydrocannabinol (THC)) and non-psychoactive (any cannabis plant with <1% THC) is defined. This non-psychoactive cannabis is categorized as a non-controlled substance and is therefore excluded from the regulation of narcotic drugs, psychotropic and other substances subject to sanitary control (schedule IIA) [8,9]. Likewise, on December 09, 2019 MINSA issued a Technical Document “Guidelines for the medicinal use of cannabis and its derivatives” [Orientaciones para el uso medicinal del cannabis y sus derivados] which is the only official reference document for physicians issued in Peru. Thus, the prescription of psychoactive cannabis must be made in the special prescription for narcotic and psychotropic drugs, and in the case of non-psychoactive cannabis it must be made in a simple prescription stored in the pharmacy [10].

Despite the existence of a regulatory framework, accessing medical cannabis in Peru remains challenging. Some perceive it as a hazardous substance leading to dependence and detrimental effects on the central nervous system. Conversely, advocates for adult or recreational use contend that its prevalence of mortality is lower compared to opioids, synthetic drugs, and even alcohol. They argue that it is a benign substance without the potential for problematic consumption. These divergent perspectives have a detrimental impact on patients, physicians [[11], [12], [13]], students [14], and the general public.

The insufficient information and knowledge among physicians may lead to prescription inhibition or misprescription, posing a challenge for the burgeoning cannabis industry [15]. Simultaneously, with the significant increase in the use of *Cannabis L.* in Peru, more patients are opting for cannabinoids to treat their ailments and illness, often without a comprehensive understanding of the fundamentals, risks, and benefits associated with its use.

This study describes the results of a survey of patients with medical cannabis use on two aspects of *Cannabis sativa L.*, such as basic knowledge, attitudes towards its use, and beliefs surrounding its regulation and safety in Peru. This study explores questions linked to the ideas constructed around cannabis in Peruvian patients in routine consultation, who have come to use it in an inopportune or prescribed way, making the results of this study important for Public Health.

## 2 Materials and methods

### 2.1 Study design, sample and inclusion criteria

This cross-sectional study was conducted at the Specialized Clinic in Therapies with Cannabinoids - CANNAVITAL (Clínica Especializada en Terapias con Cannabinoides) in Lima, Peru. The data collection was carried out between the months of January and April 2022. Employing simple random sampling from the total number of patients treated, a sample of 86 participants was obtained. The inclusion criteria were patients of all genders, aged 18 years or older, with medical prescription and active treatment with medical cannabis. The study encompassed patients using any route of cannabis administration and with a treatment duration of ≥1 year. Patients of other nationalities, with palliative cannabis use and active treatment for HIV or tuberculosis were excluded. Excluding patients of other nationalities considered potential variations in healthcare access, impacting perceptions of complementary therapies like cannabis. Furthermore, the treatment of patients with HIV or tuberculosis can significantly impact their perspectives on substance use.

Patient recruitment was executed through a multi-faceted approach. Initially, individuals from the CANNAVITAL clinic's client portfolio were contacted via email and phone calls. Additionally, patients were approached during their routine follow-up appointments, synchronized with their scheduled medical visits. This comprehensive strategy ensured a diverse and representative participant pool for our study.

### 2.2 Questionnaire and data gathering

For this study, the KUC-22 questionnaire (questionnaire on the knowledge and use of *Cannabis sativa L.*) was designed and validated. This questionnaire has 22 items with dichotomous and trichotomous responses (including a “maybe” category as a response) on the characteristics of use and knowledge of Cannabis divided into six dimensions: i) level of knowledge, ii) risk factors, iii) knowledge of legislation, iv) medical use, v) recreational use, and vi) risks of use. Only question 13 is an open-ended question on what are the main diseases treated by the use of *Cannabis sativa L.*

The validation of KUC-22 was developed by expert judgment guided by the research team of the Universidad Alas Peruanas' Graduate school. This endeavor involved the participation of 250 adult individuals from Peru, and there were no gender restrictions applied. To ensure the questionnaire's reliability, we employed a comprehensive approach. First, a global Cronbach's alpha analysis was carried out. Subsequently, we conducted an item-level analysis for each component of the questionnaire. KUC-22 has shown good internal consistency (α = 0.983) during its validation trial that also had a construct analysis through the Delphi method and expert meetings on legislation, medical use, regulation and pharmaceutical availability [16].

The surveys were administered after physical informed consent. Patients who agreed to participate in the study completed the KUC-22 in approximately 10 min. The surveys were administered in-office at the beginning of each on-site medical consultation. Each survey was reviewed for quality assurance and coded internally. The pooled data were tabulated into a data matrix in SPSS v24.0 (Armonk, US) for Windows.

### 2.3 Data analysis

The data were initially analyzed with descriptive statistics. Simple and absolute frequencies were estimated for categorical variables and mean and standard deviation for continuous variables. We used the X2 and Mann-Whitney U test to estimate differences between the KUC-22 dimensions. Also, we used Spearman's correlation coefficient considering a p-value <0.05 as statistically significant for all tests.

### 2.4 Ethical aspects

The ethical advice for this study follows the Declaration of Helsinki and the data is held in reserve private [17]. This study used printed informed consent from patients enrolled and has the approbation of the Institutional Review Board of Universidad Norbert Wiener (Reg. No. 1961–2022).

The privacy and confidentiality of participant data were diligently safeguarded in accordance with the provisions outlined in data protection law 29,733 [18]. This was achieved by anonymizing the data and employing a coding system to prevent any form of direct or indirect patient identification.

## 3 Results

A total of 88 patients consented to participate in the study. The mean age of the participants was 41 ± 7.8 years old (range 30–65 years) and 47 (53.4%) were women. All were from Lima and the time of medical cannabis use was 3 ± 1.2 years (Table 1).

**Table 1:** Baseline demographic characteristics of patients. N = 88.

Variable	Categories	*f*	%
Age group (years)	30–40	14	15.9
	41–50	48	54.6
	>50	26	29.5
Sex	Male	41	46.6
	Female	47	53.4

Sixty percent (53/88) of patients were aware of the different forms of use of cannabis and 75% (66/88) had an intuition of the risk of consumption. Sixty five percent (58/88) know that *Cannabis sativa L.* interacts with other drugs and 77.3% (68/88) of patients know that the use of cannabis depends on the quality of the product. About *Cannabis sativa L.* as a risk factor 58% (51/88) of patients know the risk for heart disease, while 79.5% (70/88) patients do not know the risk for psychiatric disease. Ninety two percent (81/88) of patients consider that, for the consumption of *Cannabis sativa L.*, prescription is necessary and 90.9% (80/88) consider that the law allows the use and abuse of cannabis (Table 2). We found a positive correlation between knowledge of cannabis and use (Rho = 0.989, p < 0.001).

**Table 2:** Inter-item analysis of knowledge of *Cannabis L.* among patients. Data in N (%).

Items	Knowledge about use of *Cannabis L.*		
	Yes	No	Maybe
Do you use your senses to detect changes and patterns of *Cannabis sativa L.* use in your environment?	18 (20.5)	26 (29.5)	44 (55)
Do you know the different forms of use of *Cannabis sativa L.*?	53 (60.2)	23 (26.1)	12 (13.6)
Do you have an intuition about the risks involved in the use of *Cannabis sativa L.*?	66 (75)	12 (13.6)	10 (11.4)
Do you know that *Cannabis sativa L.* interacts with other drugs?	58 (65.9)	12 (13.6)	18 (20.5)
Do you know that *Cannabis sativa L.* is widely available for consumption?	32 (36.4)	17 (19.3)	39 (44.3)
Do you know that the use of *Cannabis sativa L.* depends on the quality of the product?	68 (77.3)	6 (6.8)	14 (15.9)
Do you know that the consumption of *Cannabis sativa L.* is a risk factor for psychiatric disease?	11 (12.5)	70 (79.5)	7 (8)
Do you know that the consumption of *Cannabis sativa L.* is a risk factor for heart disease?	51 (58)	11 (12.5)	26 (29.5)
Do you know that the consumption of *Cannabis sativa L.* depends on the socioeconomic factor?	27 (30.7)	28 (31.8)	33 (37.5)
Do you think that the level of knowledge of law allows the use and abuse of *Cannabis sativa L.*?	80 (90.9)	6 (6.8)	2 (2.3)
Do you consider that you must present the prescription requirements for the consumption of *Cannabis sativa L.*?	81 (92)	4 (4.5)	3 (3.4)

**Fig. 1:**
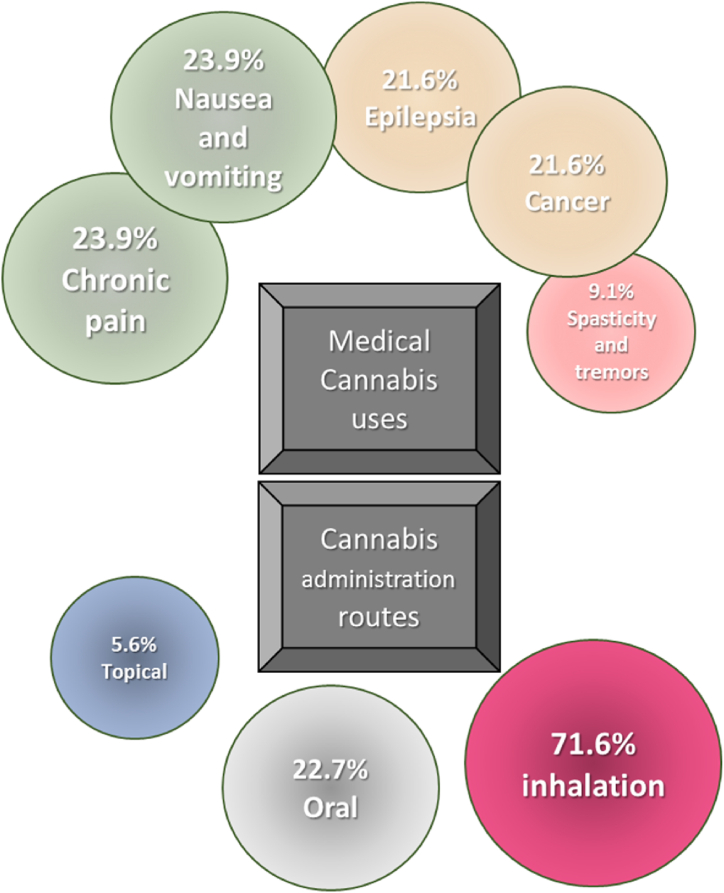
Patients' perception about the main diseases treated with medical cannabis.

**Fig. 2:**
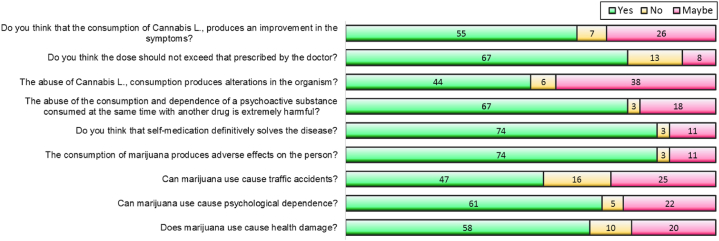
Baseline perception of Peruvian patients about the use of medicinal cannabis. All items showed significant differences (p < 0.001). Data in N (%).

Seventy one percent (63/88) of patients reported the inhalation route as the main route of *Cannabis sativa L.*, consumption, followed by the oral route (22.7%). According to patients, chronic pain and nausea and vomiting (each with 21/88, 23.9%)) are the main diseases treated with *Cannabis sativa L.*, followed by cancer and epilepsy (both with 21.6%, 19/88) (Fig. 1). Sixty two percent (55/88) of patients perceive that *Cannabis sativa L.*, produces remission of symptoms and 76.1% (67/88) consider that it should not exceed the prescribed dose. Half of the patients believe that *Cannabis sativa L.* abuse produces alterations in the organism, 76.1% (67/88) know that polydrug use has negative psychological, physical and emotional effects, 84.1% (74/88) of patients consume Cannabis L. by self-medication, and 84.1% (74/88) of patients consider that self-medication solves the disease (Fig. 2).

On the other hand, only 20.5% (18/88) of patients reported recognizing changes in consumption patterns. Also, 53.4% (47/88) of patients know that cannabis use by drivers can cause traffic accidents, 69.3% (61/88) perceive that permanent use of cannabis produces psychological dependence, and 65.9% (58/88) of patients perceive that permanent use of cannabis causes damage to health. We found differences in the answers of *Cannabis sativa L.* users in all items (p < 0.001). The inferential analysis showed among the analyzed patients a positive correlation between the perception of risk factors (Rho = 0.980, p < 0.001) and knowledge of legislation (Rho = 0.498, p < 0.001) with the use of cannabis.

## 4 Discussion

In this study, we demonstrated a high frequency of knowledge about the risks, uses and legislation of *Cannabis sativa L.,* in Peruvian patients. Patients perceive that prescription is a requirement for the consumption of *Cannabis sativa L.*, and they self-medicate as a means to solve the symptoms of their diseases. Also, the most frequent route of administration is inhalation and the main uses of cannabis are chronic pain, nausea and vomiting, and epilepsy.

### 4.1 Strengths

To the best of our knowledge, this is the first Peruvian study that evaluates the knowledge of *Cannabis sativa L.* in patients undergoing cannabinoid therapy. Another strength of this study is that a questionnaire has been designed and validated for the data collection on the use of *Cannabis sativa L.*, with which we can develop a monitoring of users during the handling of their diseases with cannabinoids during medical care.

### 4.2 Main findings

A growing body of evidence supports the global use of cannabis in patients [[19], [20], [21], [22], [23], [24], [25], [26], [27], [28], [29]]. The therapeutic potential of cannabinoids is increasingly acknowledged, as highlighted in an Italian study on cancer patients, where a majority were familiar with medical cannabis, and one-third considered its use to alleviate symptoms [23]. Recommendations for cannabinoids span various diseases and health conditions [24], with pain emerging as the primary indication for medical cannabis in around 64% of patients [23]. Recognized as beneficial, the utilization of cannabinoids for pain management has garnered substantial support in recent reviews [29]. Notably, a study involving over 2500 American patients concluded that the use of medical cannabis for chronic pain management was perceived as appropriate [20]. Our findings are consistent with this, as Peruvian patients also identified chronic pain as one of the primary conditions for which cannabinoids were used.

In another study conducted on German epilepsy patients [21], around 70% demonstrated awareness of medical cannabis as a potential treatment, expressing optimism about its efficacy in seizure control and exhibiting a keen interest in acquiring more information (∼71%). In our study, 21.6% of participants acknowledged the use of *Cannabis sativa L.* for epilepsy treatment. While the study populations differed (epilepsy patients vs. general patients), both studies underscore cannabinoids' perceived utility as therapeutic tools for effective disease management. Further research is essential to delve into patients' experiences, considering diverse cannabis doses, administration methods, and strains, given the variations in consumption across different cannabis species [30].

Furthermore, there are significant differences in accessing information about medical cannabis [31]. Our findings highlight the self-reported use of cannabis, which aligns with a study conducted on Canadian patients who reported a high prevalence of cannabis use as a substitute for other medications such as opioids, benzodiazepines, and antidepressants [20]. Similarly, a study in Germany revealed that most patients acquire knowledge about medical cannabis through the Internet rather than from physicians [21]. In an Italian study on cancer patients, it was found that while 80% of participants had heard of medical cannabis, only 2% had received information from healthcare professionals [23]. Despite the growing interest and acceptance of medical cannabis among health professionals [32,33], there remains limited knowledge regarding all aspects of its use [24,34,35]. The level of information available on cannabinoids is crucial, as it directly relates to appropriate use, as our results indicate. This is consistent with the Italian study, which found that higher education levels were associated with better understanding and use of cannabis and medical cannabis compared to patients with lower educational attainment [23]. Insufficient information can lead to misunderstandings, misconceptions about cannabinoids, and uninformed use of medical cannabis, posing ongoing challenges for the healthcare sector and policymakers involved in its regulation and use.

Regarding the legal aspects of medical cannabis use, our study revealed that a significant majority of patients (90.9%) believe that the law permits the utilization and potential misuse of *Cannabis sativa L.,* notwithstanding the acknowledged risks associated with its consumption. This finding aligns with a study involving American patients, where a majority was aware of the legal status of medical cannabis [20]. Despite the legal authorization for the use of cannabis-based medicinal products (CBMP), concerns persist regarding illegal access to these products and their quality. Notably, a substantial percentage of Canadian patients (42%) reported obtaining cannabis from illicit or unregulated sources [22], and an Australian study highlighted widespread apprehension about the uncertainty of composition and potential contamination in illicit cannabinoid products [28].

In Peru, the regulatory framework directs authorized patients to acquire cannabis from unregulated sources due to limited access to authorized products [6,9]. Our findings indicate that 77.3% of surveyed patients are aware that the efficacy of *Cannabis sativa L.* depends on the product's quality. However, the prevalence of the black market for medical cannabis compromises the benefits of CBMP, introducing uncertainty in quality, failure to meet established criteria, and potential ineffectiveness in treating various health issues. It has been shown [36] that a change in the regulation of medical cannabis improves the perception and use of medical cannabis and CBMP; therefore, it is important to review current regulations in order to improve products and quality access to cannabinoids.

Finally, the surveyed patients are aware of the risks of *Cannabis sativa L.* consumption (75%), and more than half (58%) consider it a risk factor for some diseases. These results do not agree with the findings in Italian population where only 18% consider that medical cannabis could have negative effects on their own symptoms [23]. These differences may be due to the knowledge of each group of patients, since in many countries there is still a social stigma, 18 and in others, patients still consider medical cannabis as an extract or a complete drug [21]. In this regard, it is important that more research could address the beliefs about the effects of medical cannabis on human health, since benefits have been reported for different diseases and disorders [1,37].

### 4.3 Limitations

First, it is important to acknowledge that this research is based on data collected from a single-center study conducted in Lima. Consequently, the findings may not comprehensively represent the broader spectrum of patients' perceptions and knowledge regarding *Cannabis sativa L.* within the entirety of Peru. To address this limitation and ensure a more comprehensive understanding, there is a pressing need for the development of a nationwide study. Such an undertaking would afford researchers the opportunity to explore diverse patient characteristics, investigate the specific types of cannabinoids in use, delve into the prevailing forms and dosages, and thoroughly investigate the range of medical conditions for which cannabis being utilized. Furthermore, another limitation of the study is the sample size. The pioneering nature of this investigation into patient perceptions of medical cannabis involved a relatively modest sample, consisting of 86 participants. This limited sample size can introduce potential biases and constraints in terms of the generalizability and precision of the study's findings. Third, our results showed that 92% of patients consider that a prescription is necessary for the consumption of *Cannabis sativa L.*, but did not evaluate the cost and access to medical prescription. It is important to include this variable in new research as more than half of patients have reported a cost for medical prescription to use cannabis (price ≥ USD 300) [22]. Despite these limitations, this study certainly provides new evidence on patients' knowledge of *Cannabis sativa L.*

In conclusion, our findings indicate that Peruvian patients possess a considerable understanding of the risks, applications, and regulatory framework associated with *Cannabis sativa L.* We have identified that the main use of medical cannabis is aimed at pain handling, nausea, vomiting, and epilepsy, for which patients self-medicate. Additionally, our study underscores the importance of requiring a prescription for the consumption of *Cannabis sativa L.*, highlighting an awareness of the associated risks. Based on these results, the needs, preferences and uses of patients should be better visualized by providing them with tools to improve their knowledge about cannabis, in order to avoid contradictions about whether its medicinal use is good or harmful to health. The backlash against cannabis misinformation will contribute to a more informed use, reducing misunderstandings, correcting misconceptions, and ultimately enhancing cannabinoid-based therapies for the well-being of patients.

Future research should delve into the evolving landscape of medical cannabis, considering the nuanced perspectives revealed in this single-centre study in Lima, Peru. Understanding patient knowledge, attitudes, and beliefs surrounding *Cannabis sativa L.* is vital for informing policies, guiding healthcare providers, and advancing cannabinoid-based therapies. Research into diverse cannabis consumption methods, quality considerations, and the correlation between knowledge, perception, and usage should drive future directions in optimizing medical cannabis outcomes.

## Ethics statement

This study used printed informed consent from patients enrolled and has the approbation of the Institutional Review Board of Universidad Norbert Wiener (Reg. No. 1961–2022).

## Funding statement

None.

## Data availability statement

Data associated with the study has not been deposited into a publicly available repository and data will be made available on request.

## CRediT authorship contribution statement

**José F. Ramírez-Méndez:** Writing – review & editing, Writing – original draft, Methodology, Investigation, Formal analysis, Data curation, Conceptualization. **Pedro Wong-Salgado:** Writing – review & editing, Writing – original draft, Resources, Methodology, Investigation, Formal analysis, Conceptualization. **Peter Gámez:** Writing – original draft, Supervision, Resources, Formal analysis, Data curation. **Pedro Solis:** Writing – original draft, Supervision, Methodology. **Jeel Moya-Salazar:** Writing – review & editing, Writing – original draft, Visualization, Validation, Software, Project administration, Methodology, Formal analysis, Data curation, Conceptualization.

## Declaration of competing interest

The authors declare that they have no known competing financial interests or personal relationships that could have appeared to influence the work reported in this paper.
